# Identifying *Candida albicans* Gene Networks Involved in Pathogenicity

**DOI:** 10.3389/fgene.2020.00375

**Published:** 2020-04-24

**Authors:** Graham Thomas, Judith M. Bain, Susan Budge, Alistair J. P. Brown, Ryan M. Ames

**Affiliations:** ^1^Biosciences, University of Exeter, Exeter, United Kingdom; ^2^Aberdeen Fungal Group, Institute of Medical Sciences, University of Aberdeen, Aberdeen, United Kingdom; ^3^MRC Centre for Medical Mycology at the University of Exeter, Biosciences, University of Exeter, Exeter, United Kingdom

**Keywords:** *Candida albicans*, co-expression network, network-extracted ontology, pathogen, pathogenicity genes, PEP8

## Abstract

*Candida albicans* is a normal member of the human microbiome. It is also an opportunistic pathogen, which can cause life-threatening systemic infections in severely immunocompromized individuals. Despite the availability of antifungal drugs, mortality rates of systemic infections are high and new drugs are needed to overcome therapeutic challenges including the emergence of drug resistance. Targeting known disease pathways has been suggested as a promising avenue for the development of new antifungals. However, <30% of *C. albicans* genes are verified with experimental evidence of a gene product, and the full complement of genes involved in important disease processes is currently unknown. Tools to predict the function of partially or uncharacterized genes and generate testable hypotheses will, therefore, help to identify potential targets for new antifungal development. Here, we employ a network-extracted ontology to leverage publicly available transcriptomics data and identify potential candidate genes involved in disease processes. A subset of these genes has been phenotypically screened using available deletion strains and we present preliminary data that one candidate, *PEP8*, is involved in hyphal development and immune evasion. This work demonstrates the utility of network-extracted ontologies in predicting gene function to generate testable hypotheses that can be applied to pathogenic systems. This could represent a novel first step to identifying targets for new antifungal therapies.

## 1. Introduction

*Candida albicans* is the most common cause of nosocomial fungal infections and is particularly important in vulnerable populations with suppressed immune function (Brown et al., 2012b). Mortality rates for systemic candidiasis have been estimated to be as high as 40% (Kullberg and Arendrup, 2015) and antifungal treatments for these invasive infections have only modest success in reducing these high rates (Brown et al., 2012a), despite the introduction of new antifungals, such as the echinocandins (Denning and Bromley, 2015). The discovery and development of new antifungal drugs to treat these life-threatening infections must overcome several challenges including the evolutionary similar fundamental cellular processes of humans and fungi, and the emergence of drug resistance (Denning and Hope, 2010). With high mortality rates, the emergence of drug resistance and the emergence of other pathogenic fungal species, such as *Candida auris* (Chowdhary et al., 2017), there is a drastic need for methods and targets to aid development of novel antifungal therapies.

The *C. albicans* community have employed elegant molecular and genetic approaches to identify genes involved in disease processes (Mayer et al., 2013). The key processes involved in disease are well-known and include; adhesion of yeast cells to host cell surfaces, morphological transition from yeast cells to hyphal cells, hyphal invasion by thigmotrophism, endocytosis, release of candidalysin, micronutrient scavenging, stress responses, and biofilm formation (Mayer et al., 2013; Brown A. J. et al., 2014; Brown A. J. P. et al., 2014; Moyes et al., 2016; Noble et al., 2016; Lohse et al., 2018). Indeed, targeting of these processes has been proposed as a promising strategy for antifungal development (Gauwerky et al., 2009; Vila et al., 2017). However, to identify promising targets we need a detailed understanding of these processes and, in many cases, the full complement of genes involved in these pathways is unknown. In fact, ~70% of *C. albicans* genes are uncharacterized (*Candida* Genome Database, Nov 2019) and have no experimental evidence for functional annotation (Skrzypek et al., 2016). The bioinformatic prediction of genes involved in known disease processes provides an alternative, complementary approach to potentially time-consuming experimental screens for such genes.

In the last decade, high-throughput RNA sequencing methods have empowered systems-wide studies of cellular organization and function. Methods to identify differential expression of genes (Robinson et al., 2010; Love et al., 2014) and infer gene co-expression networks (Langfelder and Horvath, 2008; Ames et al., 2013; Kramer et al., 2014) aim to predict the function of uncharacterized genes. One such method, Clique Extracted Ontology (CliXO) infers a network-extracted ontology (NeXO) from gene similarity data, such as correlated gene expression (Kramer et al., 2014). An ontology consists of entities, such as groupings or clusters of genes, that are connected by relationships. The structure is a hierarchy and provides several advantages over static network representations, as NeXOs are capable of representing the organization of biological systems on multiple scales such as pathways, complexes, and individual genes as well as capturing pleiotropy. In addition, NeXOs are data-driven and unbiased, enabling *de novo* modeling of biological processes that can generate predictions about the functions of uncharacterized genes for experimental validation. NeXOs have previously been applied to predict the functions of uncharacterized genes and identify novel functional links in yeast (Dutkowski et al., 2013). More recently, NeXOs have been used to generate hypotheses about autophagy processes implicated in disease (Kramer et al., 2017) and novel, disease-related genes in a prominent fungal phytopathogen (Ames, 2017).

In this study we have assembled publicly available transcriptomics data for *C. albicans* and use CliXO to infer a *C. albicans* NeXO that represents pathogenicity without relying on existing annotation. We demonstrate that this network is robust and can recapitulate known biological functions. By overlaying the *C. albicans* NeXO with data on characterized disease genes, we are able to make predictions about uncharacterized genes involved in various disease processes. These genes have putative roles in adhesion, hyphal development and stress responses. Furthermore, using available deletion strains we are able to phenotypically screen a subset of these candidates. Our data suggest that one such gene, *PEP8*, might play a role in hyphal development and immune evasion, which has also been suggested by other authors. Ultimately, this study demonstrates the utility of NeXOs to identify functional links between genes that can be used to generate testable hypotheses. This has allowed us to identify a potential role for a partially characterized gene in an important disease process. These tools therefore, may represent an attractive and cost effective approach to identifying virulence targets for new antifungal therapies.

## 2. Materials and Methods

### 2.1. Calculating Gene Co-expression

RNA-Seq data for *C. albicans* grown in a number of conditions, stresses and environments were downloaded from the Gene Expression Omnibus (GEO) database. These data include *C. albicans* grown in YPD and YPS (GSE41749, Grumaz et al., 2013), in planktonic and biofilm states (GSE45141, Desai et al., 2013), with a range of weak organic acids (GSE49310, Cottier et al., 2015), and in contact with host cells (GSE56091, Liu et al., 2015). In all cases all replicates, treatments and control data for strain SC5314 were used. In total, 130 RNA-Seq samples were used, as maximizing the number of samples used allows us to create the most robust NeXO possible. We note that the majority of samples were taken from an infection-related environment composed of *C. albicans* cells exposed to endothelial and epithelial cells with RNA taken at 1.5, 5, and 8 h during infection (Liu et al., 2015). Therefore, the NeXO built in this study will predominantly represent *C. albicans* infection processes related to these cell types and timepoints and is suitable for the application in this study. The addition of more expression data, from varied environments or infection stages, would allow the NeXO to represent a broader range of functions and processes.

Raw data for all publicly available data sets was re-analyzed as experimental setups and conditions may vary between sources. The *C. albicans* (strain SC5314, assembly 22, haplotype A) reference genome was downloaded from the *Candida* Genome Database (CGD, Inglis et al., 2012). RNA-Seq reads were aligned to the reference genome using bowtie (Langmead et al., 2009) and bowtie2 (Langmead and Salzberg, 2012) for single-end and paired-end reads, respectively. Alignment was performed using default parameters on a single processor and alignment files were sorted and converted to binary format using samtools (Li et al., 2009). Reads aligned to known genes were counted using the union model with HT-Seq (Anders et al., 2014) and these counts were used to estimate gene expression as reads per kilobase per million mapped reads (RPKM) with edgeR (Robinson et al., 2010). By generating estimates of expression from raw data we ensure that the data is as comparable as possible. Finally, correlations were calculated for all pairs of genes across all samples using the Pearson's correlation coefficient (PCC).

### 2.2. Generating a *C. albicans* NeXO

CliXO was used to create a *C. albicans* NeXO from correlations in gene expression (Kramer et al., 2014). CliXO requires a user-defined noise parameter α and a parameter to infer missing edges β. CliXO has been benchmarked on several 'omics data sets for the yeast *Saccharomyces cerevisiae*, including gene expression profile correlations, and it has been shown that α = 0.01 and β = 0.5 produces ontologies that are very similar to that of the Gene Ontology (GO) (Kramer et al., 2014). Therefore, in this study α and β were set to 0.01 and 0.5, respectively. Varying these parameters produces similar NeXO structures. Altering the α parameter to α = 0.02 produces a NeXO structure where entities have an average similarity to the reference *C. albicans* NeXO of 0.66 ± 0.28 (max 1.0) using the alignment and similarity metrics of Kramer et al. (2014) with a the strict hierarchy model. Varying the β parameter to β = 0.4 and β = 0.6 also produced a similar NeXO structure with average entity similarity of 0.83 ± 0.22 and 0.75 ± 0.23, respectively.

### 2.3. Aligning the *C. albicans* NeXO to the Gene Ontology

The Gene Ontology (GO) structure (OBO file) and annotations for *C. albicans* were downloaded from geneontology.org. To prepare the GO for alignment to the *C. albicans* NeXO, the structure of the GO was extracted using “is_a” GO relations, terms with no annotations to known *C. albicans* genes were removed, and redundant terms were removed. Redundant terms were defined as terms that share the same gene content as their direct descendants. When redundant terms were identified the more general term, i.e., the parent, was removed. After processing of the GO there were 776, 1,919, and 3,804 terms in the Cellular Component, Molecular Function, and Biological Process ontologies, respectively. The method of Kramer et al. (2014) was used to align the *C. albicans* NeXO to the GO ontologies using the strict hierarchy model and to generate an alignment for all nodes.

### 2.4. Scoring the Robustness of the *C. albicans* NeXO

The *C. albicans* NeXO is split into a number of entities with hierarchical relationships between them. As these entities will represent biological pathways and processes it was important to estimate the robustness of the entities with regard to the underlying experimental data used to construct the NeXO. To score the robustness of each term a measure of entity quality that considers the network support of a term and its robustness to perturbations of input data was taken from Dutkowski et al. (2013). In this measure, network support *NS*(*e*) for an entity *e* is defined as the enrichment for co-expression between genes within an entity where co-expression is defined as PCC > 0.2. Enrichment [-log(*P*-value)] is estimated using the hypergeometric distribution where the sample size is equal to all possible co-expressed genes in an entity, sample successes are the observed co-expressed genes in an entity, the population size is all possible co-expressed genes in the NeXO, and population successes are the observed co-expressed genes in the NeXO.

To quantify an entity's robustness to changes in the input data, a bootstrapping approach was employed. Samples were randomly selected with replacement to build an expression set of 130 samples. Correlations for all pairs of genes were calculated and a NeXO was inferred as described above. This process was repeated to produce 100 bootstrapped ontologies. Each bootstrapped NeXO was then aligned to the original NeXO using the method described in Kramer et al. (2014). Alignment was performed using the strict hierarchy model and all nodes were aligned. The bootstrap score *B*(*e*) for an entity (*e*) was then calculated as:

(1) $$\begin{array}{r} B\left(e\right)=\frac{1}{n}\overset{n}{\underset{i=1}{\sum }}S_{i}\left(e\right) \end{array}$$

where *n* is the number of bootstrapped NeXOs and *S*_*i*_ is the alignment score for entity *e* when the original NeXO is aligned to the *i*-th bootstrapped NeXO. Finally, the robustness score for each entity was calculated as a geometric mean of the entity's network support and bootstrap score:

(2) $$\begin{array}{r} R\left(e\right)=\sqrt{NS\left(e\right)B\left(e\right)} \end{array}$$

### 2.5. Identifying Entities Associated With Disease

Entities, clusters of genes identified from their correlated expression profiles, potentially important for disease, were identified as those containing more than two members and that show enrichment for genes implicated in disease. Two sources were used to annotate disease genes; (i) 167 disease genes identified from the pathogen-host interaction database (Winnenburg et al., 2006) and (ii) 255 genes that produce secreted and cell wall proteins thought to be associated with disease were taken from Butler et al. (2009). Fisher's exact test was used to identify enrichment by comparing the proportion of genes within each entity associated with disease to the proportion of genes in the whole NeXO associated with disease. The method of Benjamini and Hochberg (1995) was used to control for a false discovery rate of 0.05.

### 2.6. Annotating Entities in the *C. albicans* NeXO

Entities within the NeXO were annotated by identifying enriched GO terms (Ashburner et al., 2000). The GO slim ontology and annotations were downloaded from the CGD and Fisher's exact test was used to identify enriched GO terms in each entity. Briefly, for each entity each annotated GO term was tested for enrichment where the population size was the number of genes in the *C. albicans* NeXO and the sample size was the size of the entity. The population successes are the number of genes in the *C. albicans* NeXO (i.e., whole network) annotated with the specific GO term and the sample successes are the number of genes within the entity (i.e., a single cluster) annotated with that term. The method of Benjamini and Hochberg (1995) was used to control for a false discovery rate of 0.05.

### 2.7. Phenotypic Assays

Homozygous deletion strains for genes identified in entities associated with disease were assembled from Rauceo et al. (2008) and Noble et al. (2010) in VB GKO plate 1 obtained from the Fungal Genetics Stock Center (McCluskey et al., 2010). A full list of strains used in this study can be found in Supplementary File 1. Strains were stored at −80°C in 96-well plates, replicated on agar plates and subjected to a variety of phenotypic assays including; (i) thermotolerance on YPD at 25, 30, 37, and 42°C, (ii) osmotic stress with YPD+NaCl and YPD+KCl at 30°C at both 0.5 and 1 M; (iii) YPD+H_2_O_2_ at 30°C with concentrations of 2.5, 5, 7.5, and 10 mM; (iv) acidic environments from pH 4.0 to pH 7.0 at both 30 and 37°C; and (v) YPD+Fluconazole and YPD+Caspofungin at 30°C with concentrations of 1, 2 μg/ml and 0.064 and 0.128 μg/ml, respectively. Strains were incubated under these conditions for 48 h.

### 2.8. Characterization of Hyphal Growth

Hyphal growth was assessed by first examining colony morphology on agar plates containing YPD+20% serum and in Spider medium incubated at 30 and 37°C for 48 h (Liu et al., 1994). Second, overnight cultures were diluted to OD_600_, incubated in a shaking incubator in liquid YPD containing 20% serum at 37°C and cells were fixed in 10% formalin after 90 m so that hyphal growth could be observed by microscopy.

### 2.9. Macrophage Escape Assay

Bone marrow was harvested from three eight-week old male C57BL/6 mice, from which bone marrow derived macrophages (BMDMs) were differentiated, as described previously (Davies and Gordon, 2005). BMDMs were transferred to a 96-well plate at 2.4 ×10^4^ cells per well. *C. albicans* cells were grown overnight in YPD and then washed three times in phosphate-buffered saline (PBS) and resuspended in 1 ml PBS. Following counting, 7.2 ×10^4^ of *C. albicans* cells were added to each well, ensuring a 3:1 ratio of yeast to macrophage. YOYO-1 dye (150 μl) was then added to each well. Fluorescence arising from lysed macrophages was measured at 30 m intervals for 12 h in a FLUOstar Optima plate reader (BMG Labtech) using excitation 485 nm/emission 520 nm at 37°C (Bain et al., 2014).

### 2.10. Ethical Statement

BMDMs were prepared from three 8-week old male C57BL/6 mice, which were selected randomly, bred in-house, and housed in stock cages under specific pathogen-free conditions. The mice did not undergo surgical procedures prior to culling by cervical dislocation. All animal experimentation was approved by the UK Home Office and by the University of Aberdeen Animal Welfare and Ethical Review Body.

## 3. Results

### 3.1. A *C. albicans* NeXO

Expression of *C. albicans* genes from different growth states, environments, stresses, and infection models representing 130 individual samples were used to characterize the *C. albicans* system. Correlated gene expression profiles for 5,698 *C. albicans* genes were used to infer a network-extracted ontology (NeXO) with the CliXO method (Kramer et al., 2014). The resulting NeXO consists of 1,366 entities connected by 1,801 relationships where larger entities split into smaller subsets representing functions on multiple scales. The *C. albicans* NeXO structure can be found in Supplementary File 2.

**Figure 1:**
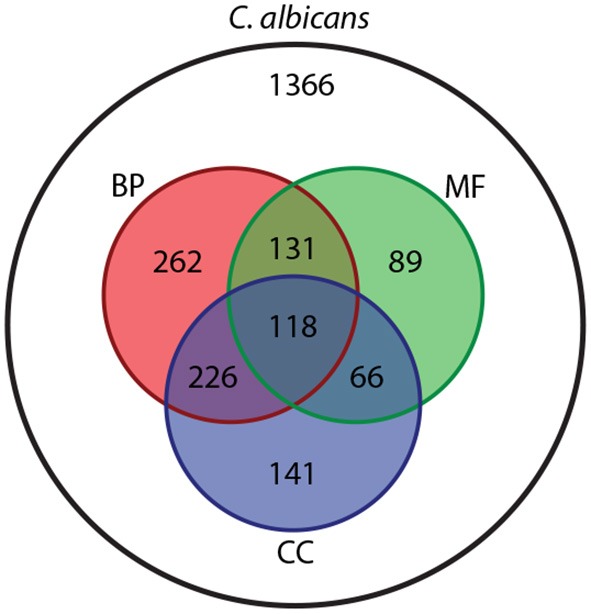
Venn diagram showing the number of entities in the *C. albicans* NeXO (white) that align to Biological Process (BP, red), Molecular Function (MF, green) or Cellular Component (CC, blue) GO ontologies.

Aligning this *C. albicans* NeXO to the GO shows that 1,033/1,366 (75.6%) entities can be aligned to at least one ontology within the GO, with many terms mapping to more than one (Figure 1). Of the 496 entities that contain more than 4 genes, 336 can be aligned to GO terms. The *C. albicans* NeXO captures 70.6% of terms in the Cellular Component ontology but only captures 18.1 and 20.9% of terms in the Biological Process and Molecular Function ontologies, respectively (Supplementary File 3). This suggests that the *C. albicans* NeXO largely describes subcellular structures and macromolecular complexes.

Aligning the *C. albicans* NeXO to the GO provides useful information about the similarity between the structure of the two ontologies. To gain more information about the function, or multiple functions, of entities within the *C. albicans* NeXO, enriched GO terms for each entity were identified using Fisher's Exact Test (*adjusted P* <0.05) and used as a functional annotation. There are 333/1,366 (24.3%) entities with significant GO term enrichment and 159/496 (32.0%) entities with significant enrichment when examining entities with greater than four genes. GO term enrichment covers terms from all three GO ontologies with 35, 20, and 23 unique GOslim terms enriched from the biological process, molecular function, and cellular component ontologies, respectively (Supplementary File 4). This suggests that our *C. albicans* NeXO focusses on a subset of functions across all GO ontologies with many entities showing no functional enrichment. These may represent noise in NeXO generation or the identification of currently unknown or unannotated pathways and functions.

To validate the *C. albicans* NeXO, robustness scores for each entity were calculated based on network support from co-expression and bootstrapping of the input expression data. We find a strong correlation between term size and robustness with larger terms being more robust (*R*^2^ = 0.87, *P* = <0.0001 on log transformed data, Figure S1). Therefore, the larger entities in the NeXO are more robust. Excluding entities with fewer than 10 members yields a mean robustness score of 4.47 compared to 0.82 when all entities are analyzed. There were two clear outliers: these were two entities immediately under the root, which both contain >5,400 genes and are far larger than any other entities in the NeXO (Figure S1), suggesting that these may be spurious entities. Overall, entities within the *C. albicans* NeXO, particularly larger entities, are robust to the underlying data suggesting that the addition or removal of data wouldn't change their composition and so they are likely to represent biological functions.

### 3.2. The *C. albicans* NeXO Contains Entities Enriched for Disease Genes

**Figure 2:**
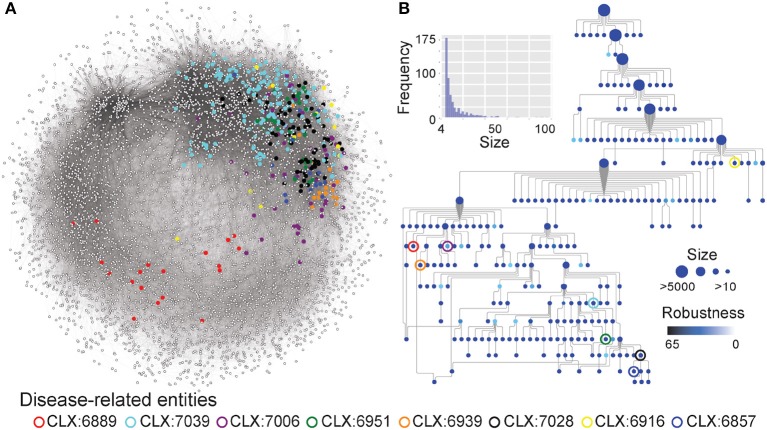
**(A)** The *C. albicans* co-expression network represented as nodes (genes) connected by edges where genes show correlated expression profiles with Pearson's Correlation Coefficient > 0.4. Entities enriched for disease genes, described in Table 1, are highlighted by colored circles and the genes that are part of these entities are also highlighted. **(B)** A network-extracted ontology (NeXO) inferred from the co-expression data, organized into entities (nodes) of genes and connected by relationships (edges). Node sizes represent the number of genes associated with each entity and only entities associated with 10 or more genes are shown. Node colors represent the robustness of the entities calculated by bootstrap analysis, where darker colors indicate a higher robustness score. Entities enriched for disease genes, described in Table 1, are highlighted by colored circles.

Although many entities in the NeXO were annotated by identifying enriched GO terms, many remain unannotated (Figure 1). The unannotated entities may represent understudied, functionally-related genes with roles in pathogenicity that are lacking GO annotation. To identify entities related to pathogenesis irrespective of GO annotation, we identified entities enriched for known disease genes in the pathogen-host interaction database (Winnenburg et al., 2006) (Figure 2). This is a major advantage of using a data-driven NeXO that can complement existing GO annotation but can also discover new entities with roles in specific biological processes.

**Table 1:** Co-expression entities enriched for known disease genes in the pathogen-host interaction database.

**Co-expression entity**	**Num genes**	**Num disease genes**	***P*_*raw*_**	***P*_*adj*_**
CLX:6916	24	5	4.92E-4	0.019
CLX:6951	33	6	2.91E-4	0.014
CLX:7006	75	9	2.55E-4	0.016
CLX:6939	30	6	1.67E-4	0.016
CLX:7039	181	14	6.16E-4	0.019
CLX:7028	139	13	1.51E-4	0.029
CLX:6857	16	4	8.97E-4	0.024
CLX:6889	19	4	0.001	0.043

*P_raw_ Fisher's exact test p-value. P_adj_ Adjusted p-value to control for a false discovery rate of 0.05 (Benjamini and Hochberg, 1995)*.

Eight entities within the *C. albicans* NeXO are enriched for known disease genes. Six of these eight entities are also enriched for the GO term “*pathogenesis*” (GO:0009405) indicating that the genes in these entities are related to disease. Additionally, several other terms related to *C. albicans* disease processes are also enriched in these entities. GO terms “*interspecies interaction between organisms*” (GO:0044419), “*filamentous growth*” (GO:0030447), and “*growth of unicellular organism as a thread of attached cells*” (GO:0070783) are all regularly enriched in putative disease-related entities. However, entities CLX:6939 and CLX:6889 show no GO enrichment and may represent areas of unstudied function associated with disease that have only been detected using this approach.

### 3.3. Entities Enriched for Known Disease Genes Predict New Candidates Associated With Disease

**Figure 3:**
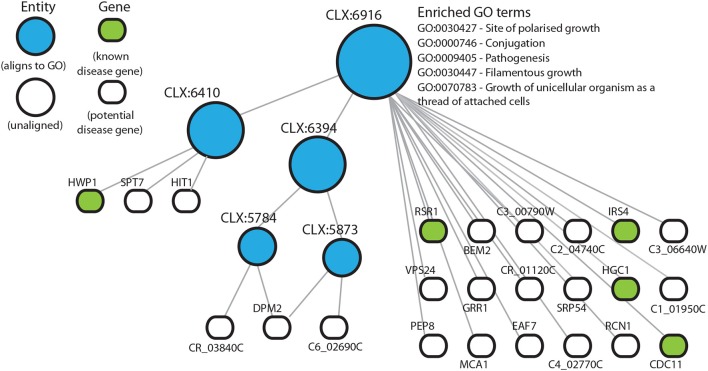
Entity CLX:6916 in the *C. albicans* NeXO is enriched for known disease genes in the pathogen-host interaction database (Winnenburg et al., 2006). The hierarchical structure shows CLX:6916 connected by edges to sub-entities (circles), and the genes (rounded rectangles) contained within the entity. Entities that are aligned to the Gene Ontology are shown in blue. Genes found in the pathogen-host interaction database are highlighted in green. Enriched Gene Ontology terms are also shown.

In those entities that are enriched for known disease genes, many other genes are uncharacterized and could potentially be involved in disease processes. In this way we can predict functional annotations for genes not present in literature-curated sources such as the GO. The entity CLX:6916 (Figure 3), contains 24 genes with 5 genes annotated as being part of the disease process in the pathogen-host interaction database (Winnenburg et al., 2006). Of the other 19 genes in this entity 6 genes have experimental annotation in the CGD and the remaining 11 genes are uncharacterized. CLX:6916 is a highly connected entity with 189 edges with a Pearson correlation coefficient >0.2, suggesting that the genes in this entity may be functionally related. The entity CLX:6916 contains genes involved in the disease process. These include *RCN1* (C6_01160W), which encodes a calcineurin-dependent signaling protein that has a role in controlling the stress response and virulence (Reedy et al., 2010). The genes VPS24 (C2_00930C) and C6_02690C have been linked to the processes of adherence Marchais et al. (2005) and hyphal growth (Bensen et al., 2004), respectively. Also, the entity CLX:6857 (Figure S2) contains the gene *TRY5* (C6_01500C), which acts as a regulator of adhesion genes (Finkel et al., 2012). These entities display tight clustering suggesting that the genes they contain are functionally related. Since these entities are enriched for known disease genes, we reasoned that the uncharacterized genes in these entities may also play roles in disease and *C. albicans* pathogenicity.

### 3.4. Entities With No Known Functional Annotation May Represent Understudied Cellular Processes

**Figure 4:**
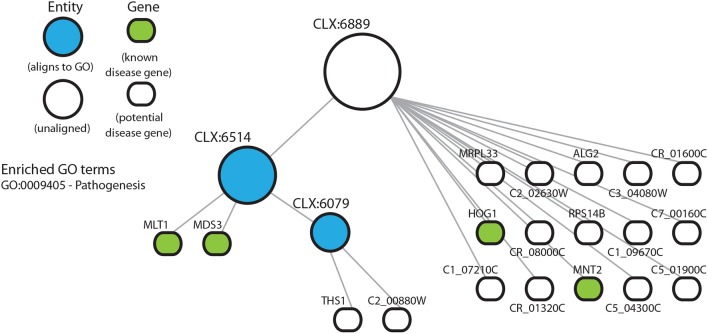
Entity CLX:6889 in the *C. albicans* NeXO is enriched for known disease genes in the pathogen-host interaction database (Winnenburg et al., 2006). The hierarchical structure shows CLX:6889 connected by edges to sub-entities (circles), and the genes (rounded rectangles) contained within the entity. Entities that are aligned to the Gene Ontology are shown in blue. Genes found in the pathogen-host interaction database are highlighted in green. Enriched Gene Ontology terms are also shown.

Entity CLX:6889 (Figure 4) shows enrichment for known disease genes but no significant enrichment for any GO terms. CLX:6889 may represent an understudied process, detectable directly from transcriptomic data using the NeXO approach, where incorrect or missing GO term annotation means we cannot detect any significant GO term enrichment or existing functional annotation. Interestingly, CLX:6939 (Figure S3) shows no GO term enrichment. However, its descendent entities, CLX:6819 and CLX:6036, are enriched for multiple GO terms. Possible interpretations again include the possibility of missing GO term annotation, or that CLX:6939 represents multiple disease-related processes with no coherent GO description while the descendent entities CLX:6819 and CLX:6036 represent singular functions with adequate GO descriptions. In these entities we find that the majority of genes annotated with GO terms have been annotated on the basis of sequence similarity or inferred from electronic annotation, with few terms annotated by mutant phenotypes or from direct assays. This suggests that the GO annotations for these genes may be inaccurate or incomplete. Indeed, ~70% of *C. albicans* genes are uncharacterized (*Candida* Genome Database, Nov 2019) and have no experimental evidence for functional annotation (Skrzypek et al., 2016). Together, the enrichment of known disease genes in this entity and the lack of experimental annotation of GO terms, may suggest that these entities, and their genes, represent coherent, but under-characterized, functional units related to disease.

The entity CLX:6889 (Figure 4) contains characterized genes involved in biofilm production (*MRPL33*—C3_01080W) (Nett et al., 2009) and response to mouse macrophages (*RPS14B*—C1_06450C) (Lorenz et al., 2004). Both of these genes have not been shown to contribute to the disease process, but have functions associated with disease. CLX:6889 also contains uncharacterized genes that are induced in response to stress (C5_04300C), involved in protein secretion (C5_01900C) and involved in the response to macrophages (THS1) (Nobile et al., 2012). These have inferred functions potentially related to disease. These entities suggest that some genes, that may have been partially characterized or annotated with GO terms, might yet have undiscovered functions involved in the disease process, which might be niche-specific, for example.

### 3.5. Phenotypic Analysis of Mutant Strains

To test whether some partially characterized genes might have undiscovered functions involved in the disease process, we selected available, homozygous null mutants corresponding to genes associated with disease-related entities in the NeXO (*AHR1, WOR2, CST20, GRR1, HGC1, HOG1, HST7, HWP1, INT1, PBS2, PEP8, RIM101, RSR1, SEF1, TRY5 HIT1, NVL6*) and irrespective of GO annotation. The mutants were subjected to a range of phenotypic assays alongside their respective control strains (CAI4+Clp10, RM1000+Clp20, SN250). These virulence-related assays included yeast-hypha morphogenesis, macrophage phagocytosis, thermotolerance, resistance to osmotic, oxidative and acid stresses, and sensitivity to the antifungal drugs, fluconazole, and caspofungin (Figure S4).

**Figure 5:**
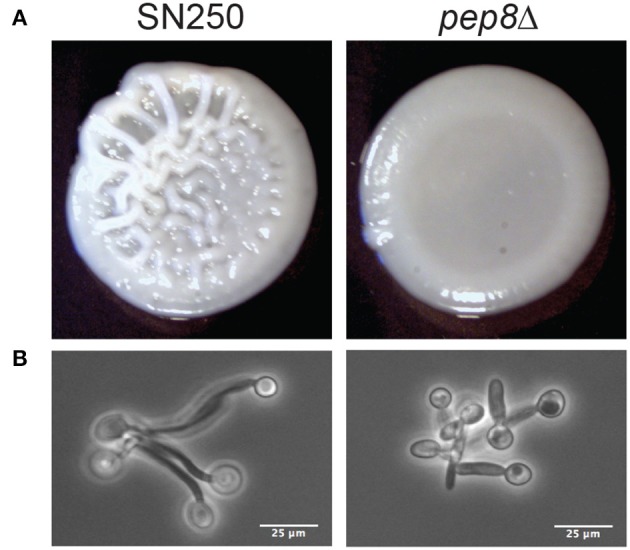
**(A)** Images of colony formation for wild-type (SN250) and *pep8* mutants grown on Spider media at 37°C for 48 h. **(B)** Evidence of reduced hyphal formation by *pep8*Δ cells compared to wild-type cells fixed in formalin at 90 m hyphal development.

There were detectable phenotypes for mutant strains in a range of conditions. Several mutants (*HIT1, NVL6, HOG1, PBS2*) showed differing levels of sensitivity to YPD+H_2_O_2_, particularly at concentrations of 7.5 and 10 mM. We also observed potential reduced growth of *hit1*Δ when exposed to fluconazole and *rsr1*Δ when treated with caspofungin. Finally, *pep8*Δ, showed an aberrant colony morphology on Spider medium (Figure 5). Whilst the control strain, SN250, displayed the distinctive wrinkly appearance associated with hyphal development, *pep8*Δ colonies were smooth, similar to the appearance of *hwp1*Δ colonies with known hyphal abnormalities, suggesting a potential defect in hyphal development (Figure S4).

### 3.6. Pep8 Promotes Hypha Formation

To explore the potential defect of *pep8*Δ cells in hyphal development, we compared their morphology to control cells (SN250) after growth in YPD containing 20% serum at 37°C (Figure 5B). The *pep8*Δ cells formed stunted hyphae compared to the control. This confirmed that the loss of Pep8 either delays or stunts hyphal development in *C. albicans*, suggesting that Pep8 plays a role in hyphal development.

**Figure 6:**
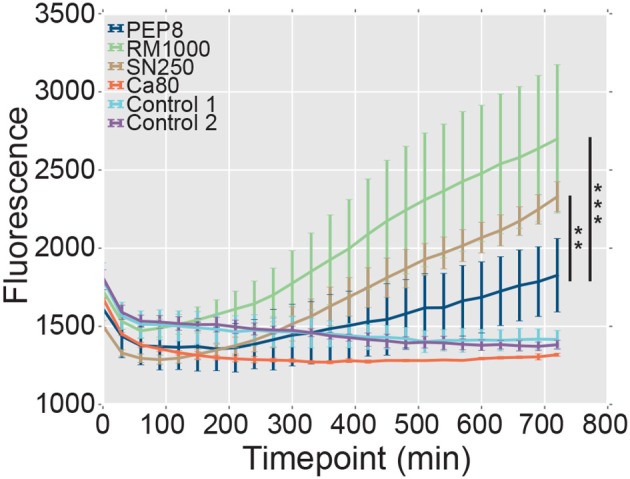
The lysis of macrophages was assayed by measuring release of the fluorescent dye, YOYO-1 (section Materials and Methods). The *pep8*Δ mutant (dark blue) displays a reduced ability to lyse and escape macrophages compared to the control wild-type strains RM1000 (green; Mann–Whitney U, ***P = 5.875e-11) and SN250 (brown; Mann–Whitney U, **P = 0.002). Uninfected macrophages (light blue, Control 1; purple, Control 2), and a hypha-deficient *cph1*Δ *efg1*Δ mutant (Lo et al., 1997) (red, Ca80) were also included as controls.

The formation of hyphae after phagocytosis is known to promote the escape of *C. albicans* cells from innate immune cells (Lo et al., 1997; McKenzie et al., 2010). Therefore, we reasoned that, given their attenuated hypha formation, *pep8*Δ cells might be less able to lyse and escape macrophages following phagocytosis. We tested this by comparing the ability of *C. albicans* strains to promote the release of the fluorescent dye, YOYO-1, from macrophages (Figure 6). Macrophages infected with wild type *C. albicans* control strains (SN250 or RM1000+CIp20) released significant amounts of the dye over the time course, whereas uninfected macrophages did not. Also, macrophages infected with the hypha-defective *C. albicans cph1*Δ *efg1*Δ mutant did not display significant levels of lysis (Figure 6), which was consistent with the role that hypha formation plays in fungal escape from macrophages (Lo et al., 1997; McKenzie et al., 2010). Significantly, the *pep8*Δ cells displayed a reduced ability to lyse the macrophages compared to the wild-type controls RM1000 (Mann–Whitney U, *P* = 5.875e-11) and SN250 (Mann–Whitney U, *P* = 0.002). However, the *pep8*Δ cells were able to escape these innate immune cells to some degree (Figure 6). This was entirely consistent with our observation that loss of Pep8 partially attenuates, but does not completely block, hypha formation (Figure 6). We conclude that Pep8 plays a role in hyphal development and defects in this gene's function affect the ability of *C. albicans* to evade the host immune system.

## 4. Discussion

Ontologies extracted from network data have been used to predict novel, disease-related genes in an important phytopathogen (Ames, 2017) and identify the function of uncharacterized genes that can be experimentally validated (Dutkowski et al., 2013). In this study, we compiled RNA-Seq data for the human fungal pathogen *C. albicans*, inferred a NeXO from correlated expression profiles, and overlaid information on known disease genes to make functional predictions for poorly- and un-characterized genes. A subset of these predictions were then tested using phenotypic screens and infection assays to identify potentially novel disease-related genes.

NeXOs have been proposed as useful tools to predict a range of cellular phenotypes and answer important biological questions (Carvunis and Ideker, 2014). These network structures have proven to be robust (Dutkowski et al., 2013), as is the *C. albicans* NeXO (Figure S1). NeXOs also recapitulate known functions; a NeXO built from a range of interaction data for *S. cerevisiae* aligns to many GO categories and best represents the Cellular Component ontology (Dutkowski et al., 2013). The same pattern can be seen in this study with many entities aligning to GO terms and best representing the Cellular Component ontology (Figure 1). Previous work looking at functional modules identified from a yeast co-expression network shows that many (>80%) Biological Process and Cellular Component terms can be represented by co-expression data (Ames et al., 2013). These results therefore suggest that the structure of NeXOs may be well suited to representing Cellular Component terms. To better represent biological processes and molecular functions, methods of generating NeXOs may have to take into account functional links between genes whose expression could be dispersed over time. Indeed, sequential activation of functionally related genes has been demonstrated during differentiation and development (Queva et al., 1998), cell wall stress in *S. cerevisiae* (Bermejo et al., 2008) and *C. albicans*-host interactions (Wilson et al., 2009).

Based on location in a NeXO and functions of characterized genes in the same entity, it is possible to generate hypotheses about the functions of poorly- or un-characterized genes. Here, we used enrichment of disease-associated genes in entities as evidence that these entities may represent disease related pathways containing as yet unknown disease genes. CLX:6916 (Figure 3) is enriched for disease-associated genes with virulence related functions, *RSR1* (CR_02140W) guides hyphal growth (Hausauer et al., 2005), *IRS4* (C3_03660W) contributes to hyphal formation (Badrane et al., 2005), *HGC1* (C1_00780C) regulates hyphal morphogenesis (Zheng et al., 2004), *CDC11* (C5_00070W) is also involved in hyphal morphology (Warenda et al., 2003) and *HWP1* (C4_03570W) encodes a major protein in the cell wall and functions in cell-surface adhesion (Staab and Sundstrom, 1998; Staab et al., 2004). This entity also contains several characterized genes that have implicated roles in disease but are not present in the pathogen-host interaction database. *GRR1* (C5_04600C) is required for cell cycle progression and involved in the negative control of pseudohyphal growth (Butler et al., 2006) and *RCN1* (C6_01160W) is a calcineurin-dependent signaling protein that controls stress response and virulence (Reedy et al., 2010). The identification of entities containing known disease genes not found in the pathogen-host interaction database highlights the reliability of these methods and also the usefulness of using pre-existing databases without the need to include expert or literature-curated knowledge. There are also several partially or uncharacterized genes, that have been linked to the disease process. For example *PEP8*, a partially characterized gene, has previously been associated with filamentation in a large-scale haploinsufficiency assay (Uhl et al., 2003).

A homozygous *pep8*Δ*/pep8*Δ deletion strain was included in the strains phenotypically assayed in this study. We find that *pep8*Δ cells produce delayed or stunted hyphae and show attenuated macrophage escape (Figures 5, 6), confirming a role for this gene in filamentation as previously reported (Uhl et al., 2003) and suggesting a role in infection. This finding also confirms recent work, which is entirely independent from this study, that has shown that *pep8* mutants demonstrate severe filamentation defects in a range of conditions (Azadmanesh et al., 2017) and these mutants abrogate azole resistance (Mount et al., 2018). Loss of function of Pep8 abrogates azole resistance by overwhelming the functional capacity of calcineurin. Interestingly, *PEP8* is clustered with *RCN1*, which encodes a calcineurin-dependent signaling protein, among other genes involved in filamentation and drug resistance. We note that not all the strains phenotypically tested appear to have roles in virulence-related processes with the recall and precision of these approaches estimated to be 0.43 and 0.31, respectively (Yu et al., 2019). However, given that NeXOs are data-driven and can be generated quickly with little expert knowledge or human intervention, they provide useful approaches for the identification of novel pathways, prediction of functions and generation of testable hypotheses, especially in those organisms that lack comprehensive annotation.

This work also demonstrates the utility of NeXOs to reveal functions at multiple levels. Entities CLX:6916 (Figure 3), CLX:6088 (Figure S2) and CLX:7006 all contain the hyphal cell wall protein Hwp1. This indicates that the *C. albicans* NeXO is capturing function at different levels and may indicate a role for Hwp1 in several disease related pathways. CLX:6088 and CLX:7006 both seem to be specifically associated with cell adhesion based on alignment to the GO and enrichment for GO terms, respectively (Supplementary Files 3, 4). These entities are children of the much larger entity CLX:7055 (2,764 members), that shows enrichment for multiple disease-related GO terms including “*pathogenesis*,” “*cell adhesion*,” and “*biofilm formation*,” and may represent a higher, or more general, description of pathogenicity. CLX:6916 appears to be associated with yeast-hypha morphogenesis and hypha function. The entity is enriched for terms relating to “*filamentous growth*” (GO:0030447), “*site of polarized growth*” (GO:0030427) and “*pseudohyphal growth*” (GO:0007124). The presence of Hwp1 in entities related to adhesion and hyphal growth tallies perfectly with the known presence of Hwp1 on the surface of hyphal cells and the protein's role in adhesion (Staab and Sundstrom, 1998; Staab et al., 1999). The ontological view allows us to identify the importance of Hwp1 in multiple-related processes that are important for pathogenesis. This is a major advantage of organizing these data into a NeXO to identify relations between entities, highlight genes present in multiple pathways or processes and find entities that represent function on different scales (Dutkowski et al., 2013).

NeXOs may also provide a tool for the functional annotation of poorly characterized genes (Dutkowski et al., 2013). The entity, CLX:6889 (Figure 4), shows an enrichment of disease-associated genes, but does not align to any term in the GO and has no enrichment for GO terms despite all genes having GO annotations. Although descendants of CLX:6889 do align to the GO and CLX:6514 is enriched for the GO term for pathogenesis (GO:0009405). The members of CLX:6889 include: *HOG1* (C2_03330C) which encodes the stress activated protein kinase involved in the core stress response (Alonso-Monge et al., 2009), *MLT1* (C1_08210C) a vacuolar membrane transporter that is needed for virulence (Theiss et al., 2002), *MNT2* (C3_01830C) a transferase with a role in adherence and virulence (Munro et al., 2005), and *MDS3* (C3_07320W) encoding a TOR signaling pathway component required for growth and hyphal formation (Davis et al., 2002; Richard et al., 2005). Other genes in this entity, both verified and uncharacterized, have roles that can be linked to disease processes. For example, a putative metallodipeptidase (C5_04300C) is a target gene of a small network of transcription regulators that control biofilm development (Nobile et al., 2012) and is targeted for sumoylation, where sumoylation targets have been shown to have roles in cell cycle progression and stress responses (Leach et al., 2011). The genes in this entity have varied roles and seem loosely to be related to growth and its regulation. Although this entity might be produced by a random clustering of genes, the enrichment for disease-associated genes and functions assigned to the entity's descendents suggests CLX:6889 may represent a true functional module related to disease not described by current GO annotations. Indeed, most of the GO annotations for genes in this entity are inferred from electronic annotation rather than direct experimental evidence and previous work using network extracted ontologies has shown that entities that do not align to the GO can represent true biological modules (Dutkowski et al., 2013). The genes in this entity therefore, may represent new targets for functional characterization related to pathogenesis.

In this study, we use a *C. albicans* NeXO to generate testable hypotheses about this important fungal pathogen. It is important to note that the underlying data used to infer NeXOs is crucial to the types of questions that can be answered. The *C. albicans* NeXO, leverages publicly available data and is largely constructed from infection-related data (see section Materials and Methods), making it well suited to generating hypotheses about infection-related processes. This may be why the NeXO is useful to identify groups of genes related to hyphal development and similar pathogenic processes. However, we note that other processes are clearly represented by the *C. albicans* NeXO, with 24% of entities enriched for GO terms across all 3 Gene Ontologies and only 2~% of entities enriched with the “*pathogenesis*” GO term. Previous studies have used NeXOs to describe broad functional categories for the yeast *S. cerevisiae* and generate hypotheses about the function of uncharacterized genes (Dutkowski et al., 2013). This was made possible by utilizing integrated data from protein-protein interactions, gene co-expression and genetic interactions, which represent varied areas of biological function (Ames et al., 2013). Therefore, the addition of further data, both for different infection scenarios and environments, would produce a *C. albicans* NeXO that represents more varied areas of biological function and may well increase alignment to the GO. Likewise, the addition of known disease genes from the literature to supplement the pathogen-host interaction database would increase our set of known disease genes and may well allow the identification of further disease processes in the NeXO. Nevertheless, the data used in this study have been able to recapitulate known functions (Figure 1) and generate hypotheses for the role of several uncharacterized genes in disease processes (Figures 3, 4, Figures S2, S3). Even though only a handful of genes predicted to be involved in disease were available from the FGSC, we were still able to identify *PEP8* as having a potential role in hyphal development and immune system evasion.

## 5. Conclusion

Here, we have built a NeXO for *C. albicans*, leveraging publicly available data, to identify novel gene networks involved in pathogenicity. We have shown that the NeXO is robust and recapitulates known biology by aligning to the GO. By identifying enrichment of known disease genes in entities we make predictions about pathways and partially or uncharacterized genes potentially involved in pathogenicity. One such gene, *PEP8*, is shown to produce stunted or delayed hyphae and attenuated immune system evasion. This work, along with that of others, suggests further characterization of *PEP8* would be important for a more detailed understanding of infection processes. This study, therefore, shows the utility of NeXOs for pathway identification, functional annotation, and hypothesis generation that can be applied to multiple systems and processes.

## Data Availability Statement

The publicly-available datasets analyzed for this study can be found in the Gene Expression Omnibus (IDs: GSE41749, GSE45141, GSE49310, GSE56091). The network-extracted ontology generated as part of this study is available in the Supplementary Information.

## Author Contributions

RA and AB contributed conception and design of the study. RA performed the computation analysis and wrote the first draft of the manuscript. RA, JB, and SB performed the experimental analysis. GT, JB, and AB wrote sections of the manuscript. All authors contributed to manuscript revision, read, and approved the submitted version.

## Conflict of Interest

The authors declare that the research was conducted in the absence of any commercial or financial relationships that could be construed as a potential conflict of interest.
