# Supplementary material for: Identifying Candida albicans Gene Networks Involved in Pathogenicity
Source: Front Genet. 2020 Apr 24;11:375. doi: 10.3389/fgene.2020.00375 (PMC7193023; doi:10.3389/fgene.2020.00375)
Supplement: Supplementary file 5 [file Data_Sheet_1.pdf]

Supplementary Material

1 SUPPLEMENTARY FILES

Supplementary File 1.

SuppFile1-strains.xlsx. List of strains used in this study. This table contains information on the deletion strains used in this study including the source and disease entities in the *C. albicans* NeXO in which they are present.

Supplementary File 2.

SuppFile2-NeXO.xlsx. The structure of the *C. albicans* NeXO. Columns 1 (parent) and 2 (child) represent the entity ID (numerical value) for internal nodes or gene ID (CAL format from the Candida Genome Database) for terminal nodes connected by a parent-child relationship. Column 3 identifies the type of relationship, with 'default' representing an internal to internal node relationship and 'gene' representing an internal node to terminal node relationship. Column 4 is the distance between the nodes represented by half the difference in entity weight between the parent and child, where entity weight is the similarity value at which the term was inferred.

Supplementary File 3.

SuppFile3-alignments.xlsx. Alignment of the *C. albicans* NeXO to the Gene Ontology. Columns show the *C. albicans* entity, the aligned GO term, alignment score, the namespace and the GO term description. There is a single sheet for the 'Biological Process', 'Molecular Function' and 'Cellular Component' namespaces.

Supplementary File 4.

SuppFile2-enrichment.xlsx. Gene Ontology term enrichment for all entities identified in the *C. albicans* NeXO. Columns show the GO term, description, namespace, P-value and corrected P-value for all enriched terms.

2 SUPPLEMENTARY TABLES AND FIGURES

2.1 Figures

REFERENCES

Winnenburg, R., Baldwin, T. K., Urban, M., Rawlings, C., Köhler, J., and Hammond-Kosack, K. E. (2006). Phi-base: a new database for pathogen host interactions. *Nucleic acids research* 34, D459–D464

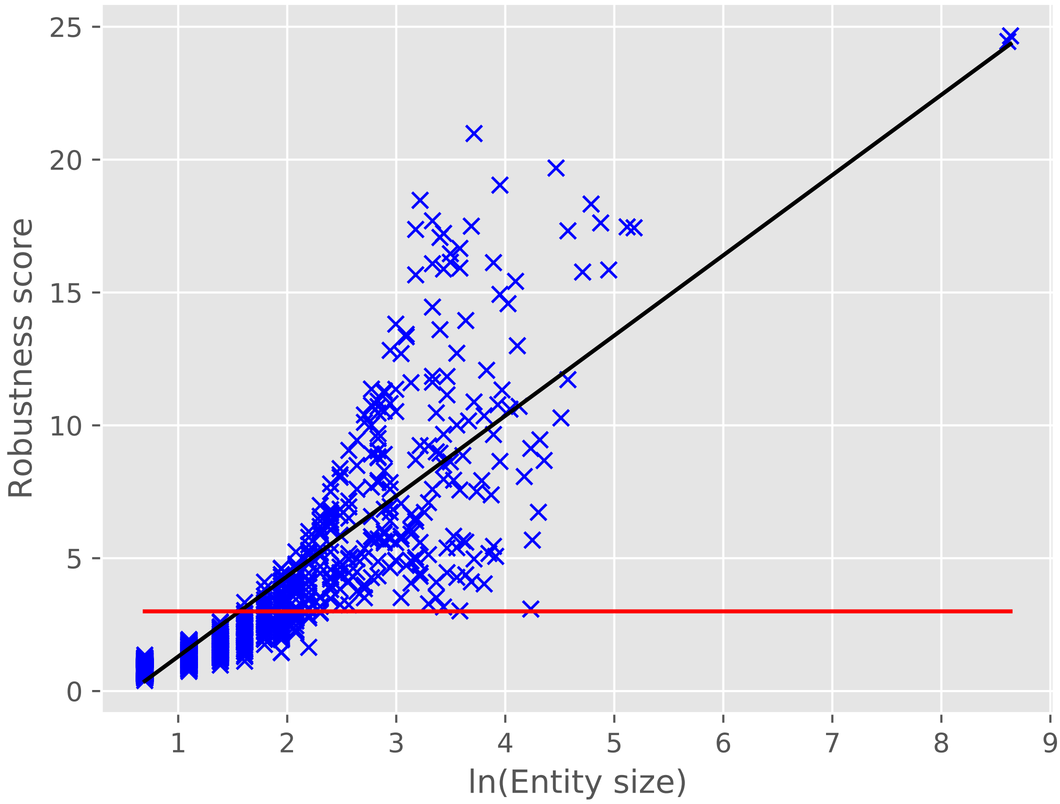

Figure S1. Robustness is calculated based on network support from co-expression data and from bootstrapping the input data. Entity sizes are normalised using the natural logarithm. The red line marks the median entity robustness score (3.0) for all entities from the *C. albicans* NeXO that align to the Gene Ontology. There is a strong positive correlation between term size and robustness (R^2 = 0.87, P = <<0.0001).

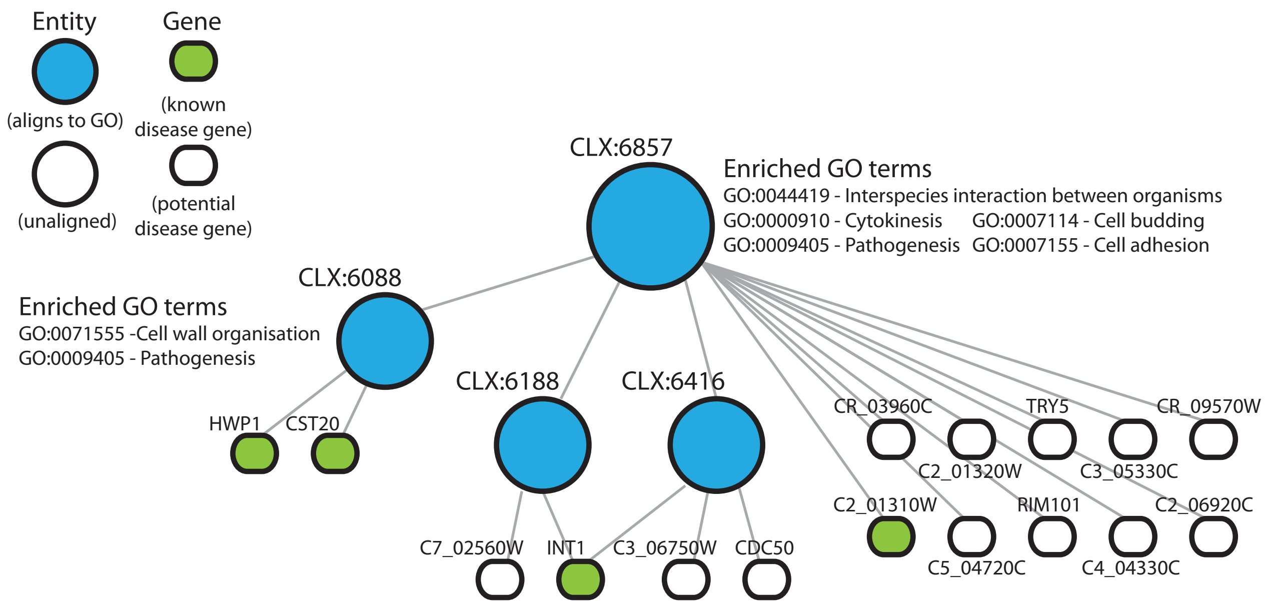

Figure S2. Entity CLX:6857 in the *C. albicans* NeXO is enriched for known disease genes in the pathogen-host interaction database (Winnenburg et al., 2006). The heirarchical structure shows CLX:6857 connected by edges to sub-entities (circles), and the genes (rounded rectangles) contained within the entity. Entities that are aligned to the Gene Ontology are shown in blue. Genes found in the pathogen-host interaction database are highlighted in green. Enriched Gene Ontology terms are also shown.

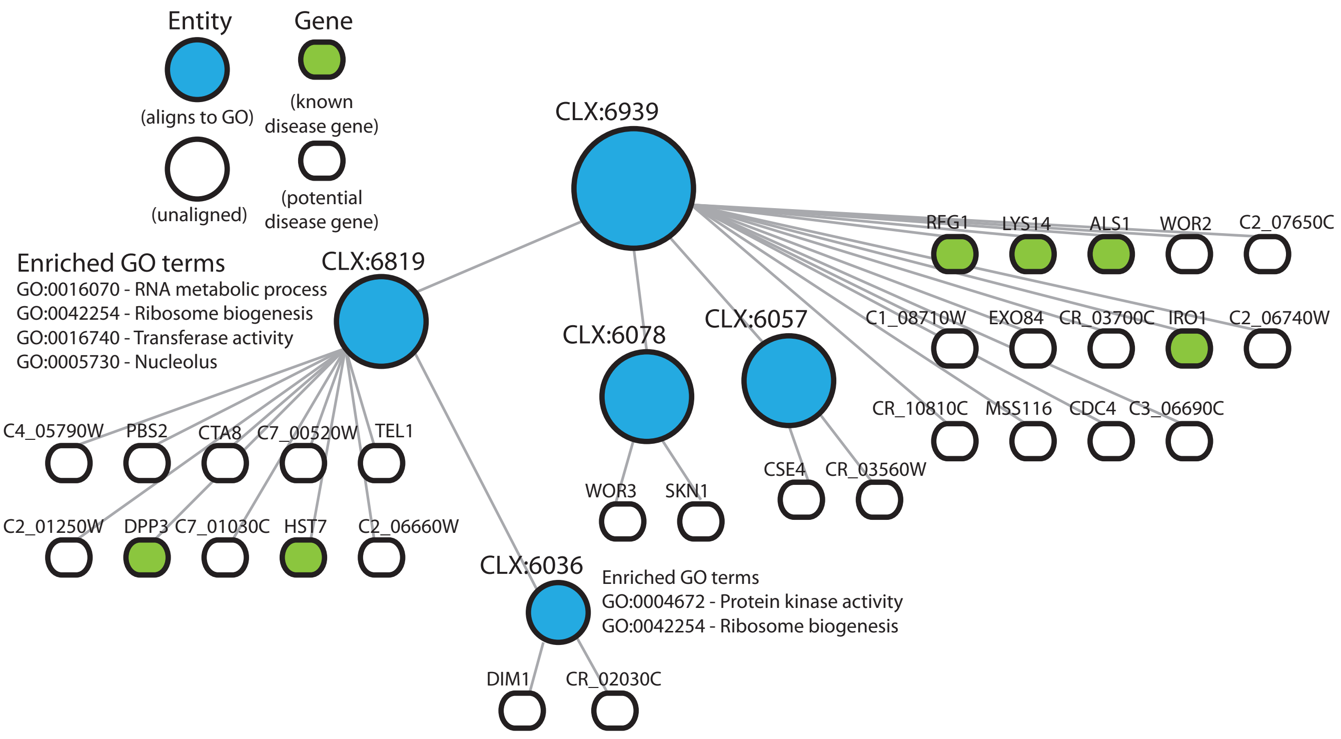

Figure S3. Entity CLX:6939 in the *C. albicans* NeXO is enriched for known disease genes in the pathogen-host interaction database (Winnenburg et al., 2006). The heirarchical structure shows CLX:6939 connected by edges to sub-entities (circles), and the genes (rounded rectangles) contained within the entity. Entities that are aligned to the Gene Ontology are shown in blue. Genes found in the pathogen-host interaction database are highlighted in green. Enriched Gene Ontology terms are also shown.

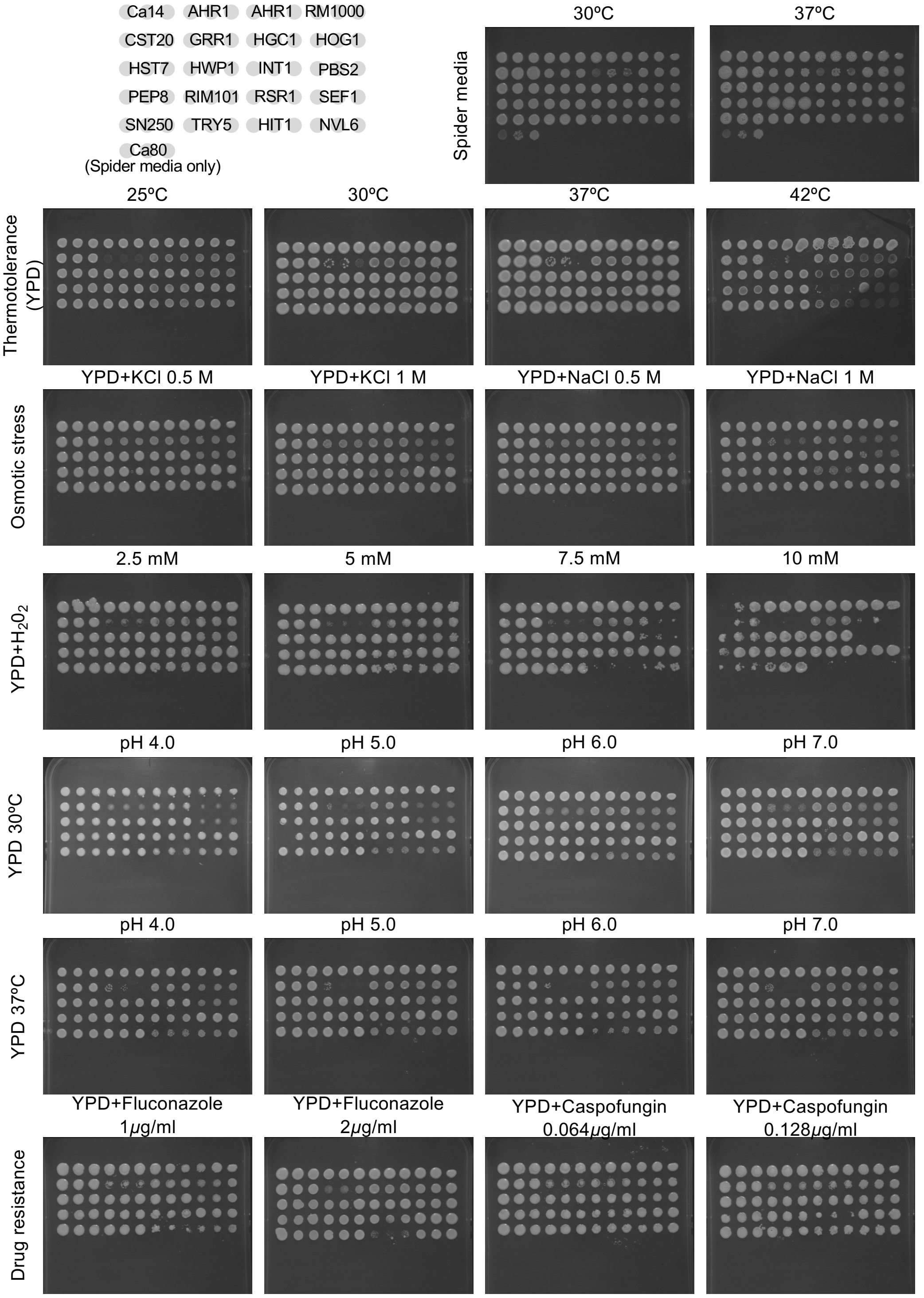

Figure S4. Phenotypic assays of homozygous null mutants *AHR1, WOR2, CST20, GRR1, HGC1, HOG1, HST7, HWP1, INT1, PBS2, PEP8, RIM101, RSR1, SEF1, TRY5 HIT1, and NVL6*, and control strains (CAI4+Clp10, RM1000+Clp20, SN250 and Ca80 (spider media only)). The conditions include; thermotolerance on YPD at 25°C, 30°C, 37°C and 42°C, osmotic stress with YPD+NaCl and YPD+KCl at 30°C at both 0.5 M and 1 M; YPD+H_2O_2 at 30°C with concentrations of 2.5 mM, 5 mM, 7.5 mM and 10 mM; acidic environments from pH 4.0 to pH 7.0 at both 30°C and 37°C; and YPD+Fluconazole and YPD+Caspofungin at 30°C with concentrations of 1 μg/ml, 2 μg/ml and 0.064 μg/ml and 0.128 μg/ml, respectively.
